# Orthopaedic Surgery in Times of COVID-19 in India

**DOI:** 10.5704/MOJ.2007.005

**Published:** 2020-07

**Authors:** PK Majumdar, RK Gupta

**Affiliations:** Department of Orthopaedics, Pandit Bhagwat Dayal Sharma Post Graduate Institute of Medical Sciences, Rohtak, India

Dear Editor,

We are used to invisible enemies in our practice but this time it is different. We may be the victims this time, like other orthopaedic surgeons around the world, along with our patients, despite meticulous precautions. In these testing times, as we enter phase III of the coronavirus pandemic in India, we are faced with difficult decisions to make regarding the surgeries to perform and those to defer^1, 2^.

The fear of asymptomatic carriers in patients and colleagues should not weigh on the decision to operate but should be evaluated by the urgency of the procedure; existing and anticipated COVID-19 cases in the hospital and region; availability of PPE, beds and staff; and finally, age and health of the patient.

What constitutes elective surgery? The traditional elective surgeries like arthroscopy and arthroplasty are obvious no-brainers but what about trauma? Are all fresh fractures emergencies or only life and limb saving surgeries? This is tight rope walking for surgeons.

In times where several hospitals have started taking special ‘Corona consent’, do we save ourselves and hopefully patients’ lives now and manage complications like non-union or malunion later^3^? How about our relatives and patients with whom we have a long-standing or good relationship with? How do we turn them down? The tough task of decision making should be a collective effort after discussion of each case and cannot be just put into one category or other. Luckily the lockdown imposed by the government has considerably decreased the number of trauma patients but on the flip side, being in an apex institute means every case gets referred to us from the smaller hospitals/nursing homes that have closed doors to patients they usually cater to otherwise. In our humble opinion, upper limb surgeries take a back seat especially clavicle, scapula, diaphyseal upper limb as well as non-dominant hand fractures. Femur fractures need to be addressed while patella, leg, foot and non-life-saving pelvi-acetabular surgeries may be delayed or managed conservatively. Spine fractures may be managed conservatively if cauda equina symptoms or significant deficit is not present. Pediatric and congenital deformity surgeries may be postponed whereas, malignancies, tendon injuries, amputations, acute infections and abscesses of bones or joints, periarticular fractures and periprosthetic lower limb fractures may not wait. Outpatient visits may be restricted to recent postoperative patients only.

Senior surgeons or those with co-morbidities may minimise patient interaction especially in operation theatres and allow essential surgeries to be performed by younger surgeons. The lockdown has thrown outpatients into disarray as public transport is not available and most times, no direct telephone access to orthopaedic surgeons may be possible in certain situations. Those with plaster or acrylic casts on, well beyond two months, have no way to cut them at home and no means to reach the hospital.

There are many questions, yet few answers. In the end it is an occupational hazard that we have to live with.

Note: The first author had a recent uncomplicated right-side anterior shoulder dislocation (dominant) with a large labral tear apart from a ligament injury, yet chose to avoid surgery now despite having access to the best surgeons in the country, till the air clears and hopefully not have lasting worries.
