# Supplementary material for: Orthopaedic Surgery in Times of COVID-19 in India
Source: Malays Orthop J. 2020 Jul;14(2):151–3. doi: 10.5704/MOJ.2007.005 (PMC7513648; doi:10.5704/MOJ.2007.005)
Supplement: Related texts

# Reply: Response from Editor

**Published:** 2020-07

**Authors:** Ismail Hadisoebroto Dilogo

**Affiliations:** Department of Orthopaedic and Traumatology, Faculty of Medicine Universitas Indonesia-Cipto Mangunkusumo Hospital, Jakarta, Indonesia

**Covid-19 in Indonesia: Lesson from our patients**

“The new normal” is a phrase that has been repeatedly used to depict our daily situation. COVID-19 pandemic has forced us to adapt out of norms indefinitely, as the discovery and investigation of vaccine and definitive treatment are still along the way. In Indonesia, the rate of confirmed cases and death tolls keep increasing, as the tested population have yet reached 1% of total national population. And sadly, we also have one of the highest death tolls of healthcare workers in Southeast Asia^1^.

American College of Surgeons recommends hospitals to limit the group of cases that shall be operated^2^. In one of Indonesian public hospitals, Orthopaedic surgeons are only allowed to perform surgeries on emergency, malignancy, and infection cases, whereas elective surgeries shall be postponed indefinitely. For private practices, implication of such rule may put the hospitals in bankruptcy, as most of their funding rely on the number of patients visit, whereas their expenditures may be higher. Hence, this case restriction is not applicable for private hospitals and elective patients are still receiving care.

Hospital visits shall be limited to lessen the exposure. To lessen the number of visits, some Orthopaedic surgeons extend the length of post-operative joint replacement stay of their patients up to 10-14 days to receive the multidisciplinary care, including rehabilitation and wound care. Following their discharge from the hospital, patients may consult their surgeon through telemedicine unless emergency cases ensue.

Acknowledging the Covid-19 infection status of the patient and applying risk stratification system may answer these problems. Initial assessment (clinical, laboratory, and radiology) and COVID-19 testing by PCR or serology prior to surgery planning are needed to stratify patients’ risks. The origin and quality of sample tested for PCR determines the success rate of COVID-19 detection. A study from Wang *et al* shows the differences in positive PCR rates among all origin of human biologic samples, revealing the highest positive rate is taken from bronchoalveolar lavage fluid (93.%), nasal swabs 63% and pharyngeal swabs 32%^3^.

Risk stratification allows physicians to ensure the safety of patients and hospital workers from any cross infection. Patients with higher risk of COVID-19 infection must comply to the designated zone, with workers donned with appropriate PPE. In any procedural room, including the operating theatre, hospital must assess for any possible aerosol-generating procedures and later, provide the appropriate PPE. This system can also be applied to a bigger community to avoid large scale lockdown or movement restriction, which will put the nation’s economy in danger.

As take-home messages, patients shall reduce their number of visits to the hospital and utilise telemedicine for consultation and extension of prescription, if necessary. To support this measure, attendings may extend their length of stay for postoperative patients to receive comprehensive care. However, acknowledging patients’ status of COVID infection plays important role in determining subsequent care of patient.

## References

[1] Chew A (2020). Coronavirus: Indonesia sees cases surge as death toll among doctors mounts.. South China Morning Post..

[2] ACS COVID-19: Recommendations for Management of Elective Surgical Procedures.. American College of Surgeons..

[3] Wang W, Xu Y, Gao R, Lu R, Han K, Wu G (2020). Detection of SARS-CoV-2 in Different Types of Clinical Specimens.. JAMA..

# Reply: Response from Editor

**Published:** 2020-07

**Authors:** Koh Joyce SB, Tan Mann Hong, Howe Tet Sen

**Affiliations:** Department of Orthopaedic Surgery, Singapore General Hospital, Musculoskeletal; Academic Clinical Program, Singhealth Duke-NUS AMC. Academia Level 4, 20 College Road, Singapore 169856

**Adequate safety, appropriate care: Operating in Orthopaedics during COVID-19**

In Singapore, the Orthopaedic community share your concerns where healthcare personnel are at increased risks. Evidence of COVID-19 transmission from asymptomatic individuals^1^ brings challenges to orthopaedic practice in terms of nosocomial transmission, isolation and cohorting requirements and increased post operative complications in undetected individuals.

It is imperative healthcare personnel, the very resource indispensable in our fight against COVID-19, do not fall victim themselves. Nosocomial transmission rates can be minimised by screening, protection and minimising contacts. Screening includes obtaining relevant contact histories, coryzal symptoms, and identifying at-risk cohorts such as institutionalised patients and workers residing in dormitories in Singapore’s context. All individuals, including healthcare workers and patients are mandated to wear masks and good hand hygiene practices are performed universally. In treating established COVID-19 cases and suspect cases, especially for procedures with aerosolization^2^, full personal protective equipment including fitted n95 masks, goggles, face-shields, gown and gloves are donned and appropriately discarded. Safe distancing, lock-downs and circuit breakers imposed by governments world-wide serve to minimise person-to-person contact, and the healthcare institution responds by limiting consults and surgeries to urgent conditions.

When Singapore went into DORSCON Orange on 7 Feb 2020, non-urgent surgeries were postponed to prevent overwhelming the healthcare system, and allow for effective cohorting and isolation practices with a reduced inpatient load. However, with the realisation that asymptomatic carriers can still have stormy post-surgical recoveries^3^, the risk-benefit ratio needs to be properly weighted in decisions to undertake any Orthopaedic surgery.

Instead of urgent versus non-urgent procedures, we should ask ourselves if the pathology is life-threatening, limb threatening or life-style threatening. In instances of life or limb threatening conditions such as major musculoskeletal sepsis, open injuries, and acute spinal cord compression, the benefits of intervention would outweigh the impact of COVID-19 on post-surgical recovery. In other instances, urgent surgeries such as hip fractures, and musculoskeletal oncology would need to take into account current risks, the capability of the healthcare infrastructure to holistically deliver the care required, and the potential length and effects of a delay. The answer is not obvious, as the need to undertake a short delay becomes necessary depending on how overwhelmed the healthcare situation in a country is. We agree with the author that conservative management should be undertaken where outcomes are not compromised. Arthroplasty and most arthroscopic procedures are currently deferred. However, with a protracted pandemic, frustrated patients and overbooking of downstream surgical lists are becoming a concern in Singapore. The embargo on elective procedures during the Circuit Breaker period in Singapore has financial implications on private surgeons. The public sector is less affected financially, but is subject to frequent manpower deployments.

Till a safe and effective vaccine becomes readily available, the COVID-19 pandemic could change surgical protocols forever. As elective work resumes, screening practices should continue and universal precautions rigorously followed. We should continue to study best practices, such as the routine swabbing of pre-surgical patients^4^, anticoagulation in view of possible thrombotic tendencies^5^, and remain open to adopting them in practice.

## References

[1] Furukawa NW, Brooks JT, Sobel J (2020). Evidence Supporting Transmission of Severe Acute Respiratory Syndrome Coronavirus 2 While Presymptomatic or Asymptomatic.. Emerg Infect Dis..

[2] Anderson EL, Turnham P, Griffin JR, Clarke CC (2020). Consideration of the Aerosol Transmission for COVID-19 and Public Health.. Risk Anal.

[3] Lei S, Jiang F, Su W, Chen C, Chen J, Mei W (2020). Clinical characteristics and outcomes of patients undergoing surgeries during the incubation period of COVID-19 infection.. EClinicalMedicine..

[4] Zizzo M1, Bollino R2, Annessi V3 (2020). Pre- and post-operative screening in limited-term elective cancer surgery patients during the COVID-19 pandemic.. J Visc Surg..

[5] Violi F, Pastori D, Cangemi R, Pignatelli P, Loffredo L (2020). Hypercoagulation and Antithrombotic Treatment in Coronavirus 2019: A New Challenge.. Thromb Haemost..
